# Development of a checklist for evaluation of shared decision-making in consultation for extremely preterm delivery

**DOI:** 10.1038/s41372-024-02136-6

**Published:** 2024-10-22

**Authors:** Michael Guindon, Dalia M. Feltman, Carrie Litke-Wager, Elizabeth Okonek, Kaitlyn T. Mullin, Uchenna E. Anani, Peter D. Murray II, Christopher Mattson, Jeanne Krick

**Affiliations:** 1 Department of Pediatrics, Division of Neonatology, Brooke Army Medical Center, San Antonio, TX USA; 2 Department of Pediatrics, Uniformed Services University of the Health Sciences, Bethesda, MD USA; 3 Department of Pediatrics, NorthShore University Health System, Evanston, IL USA; 4 Department of Pediatrics, Division of Neonatology, Vanderbilt University Medical Center, Nashville, TN USA; 5 University of Virginia School of Medicine, Charlottesville, VA USA; 6 Department of Pediatrics, Division of Critical Care Medicine, Ann and Robert H. Lurie Children’s Hospital of Chicago, Chicago, IL USA

**Keywords:** Paediatrics, Patient education, Medical ethics

## Abstract

**Objective:**

Shared decision-making (SDM) between parents facing extremely preterm delivery and the medical team is recommended to develop the best course of action for neonatal care. We aimed to describe the creation and testing of a literature-based checklist to assess SDM practices for consultation with parents facing extremely preterm delivery.

**Study design:**

The checklist of SDM counseling behaviors was created after literature review and with expert consensus. Mock consultations with a standardized patient facing extremely preterm delivery were performed, video-recorded, and scored using the checklist. Intraclass correlation coefficients and Cronbach’s alpha were calculated.

**Result:**

The checklist was moderately reliable for all scorers in aggregate. Differences existed between subcategories within classes of scorer, and between scorer classes. Agreement was moderate between expert scorers, but poor between novice scorers. Internal consistency of the checklist was excellent (Cronbach’s alpha = 0.93).

**Conclusion:**

This novel checklist for evaluating SDM shows promise for use in future research, training, and clinical settings.

## Introduction

Parents facing extremely preterm delivery (before 25 weeks gestation) partner with neonatologists and obstetricians to make decisions for the care of their children. Antenatal counseling in the setting of extreme prematurity serves to provide information to those parents to support them in making resuscitation choices for their infant [1]. The key components for counseling a parent facing extremely preterm delivery vary based on the individual circumstances surrounding the pregnancy and expected delivery. How these components are addressed during the consultation may change depending on patient characteristics, health professional preferences, and clinical circumstances surrounding the encounter [1–7]. Sometimes the topics that seem essential to the counseling physician may be irrelevant, at best, or harmful, at worst, in the view of the expectant parent. As such, no single approach to antenatal counseling will be effective for all families. Shared decision-making (SDM) is therefore recommended to facilitate integration of parental values with the medical knowledge and experience of physicians during the antenatal consultation.

While widely accepted as the appropriate approach to making difficult decisions with unclear consequences, SDM is difficult to define and enact in routine practice [8, 9]. Implementation of SDM presents a unique set of challenges in counseling for extremely preterm delivery, including uncertainty about survival and long-term neurodevelopmental outcomes, need for emergent delivery precluding consultation, environmental factors such as hospital resources, and healthcare professional attitudes toward SDM [1, 4, 7, 10]. Discussions may also be shaped more by local policy than patient characteristics. Many institutions have implemented formal policies regarding antenatal counseling; however, the evidence for how to ensure that SDM between physicians and expectant parents occurs during these consultations remains weak [3, 7, 11–13]. Further complicating this practice is the variability of education and training that neonatologists undergo regarding antenatal counseling in general [10, 14, 15]. Training in communication has been reported by neonatal-perinatal fellows to be a priority, but often not given much dedicated time in a busy didactic calendar [10].

Little is known about how neonatologists perform SDM in antenatal counseling for extremely premature deliveries. Counseling tools often target the provision of information rather than the more holistic the process of SDM. Instruments that have been used to assess the process of SDM have previously been found to lack evidence regarding measurement quality [16]. Several tools have been proposed to aid in the assessment of SDM by health professionals and patients, but these have largely been unvalidated for use in counseling parents facing extremely preterm delivery [17–19]. Information on what components of SDM are important to parents facing extremely preterm birth are lacking, in part due to the challenges of assessing components of SDM in this setting.

Several approaches exist for investigating the validity of an assessment. A dominant framework that is commonly cited is that described by Messick [20]. This framework, which defines five sources of validity evidence for assessment, was utilized in the development of the checklist. The purpose of this study was to develop and test a checklist to assess discrete components of SDM during antenatal consultation encounters for parents facing extremely preterm delivery.

## Methods

### Checklist development

This SDM checklist was developed and assessed for validity using Messick’s framework [20]. This framework evaluates five sources of validity evidence: content, response process, internal structure, relations to other variables, and consequences. Specifically, this phase of the checklist development and testing focused on the first three sources of validity evidence. To address the content source of validity, a review of the literature was performed to understand what is known about SDM for neonates born extremely premature. A search of the literature was performed using the PubMed database for English language publications from 2009 to 2019 with search terms “shared decision making + neonatology;” “shared decision making + obstetrics + prematurity;” and “shared decision making + obstetrics + periviable.”

**Fig. 1:**
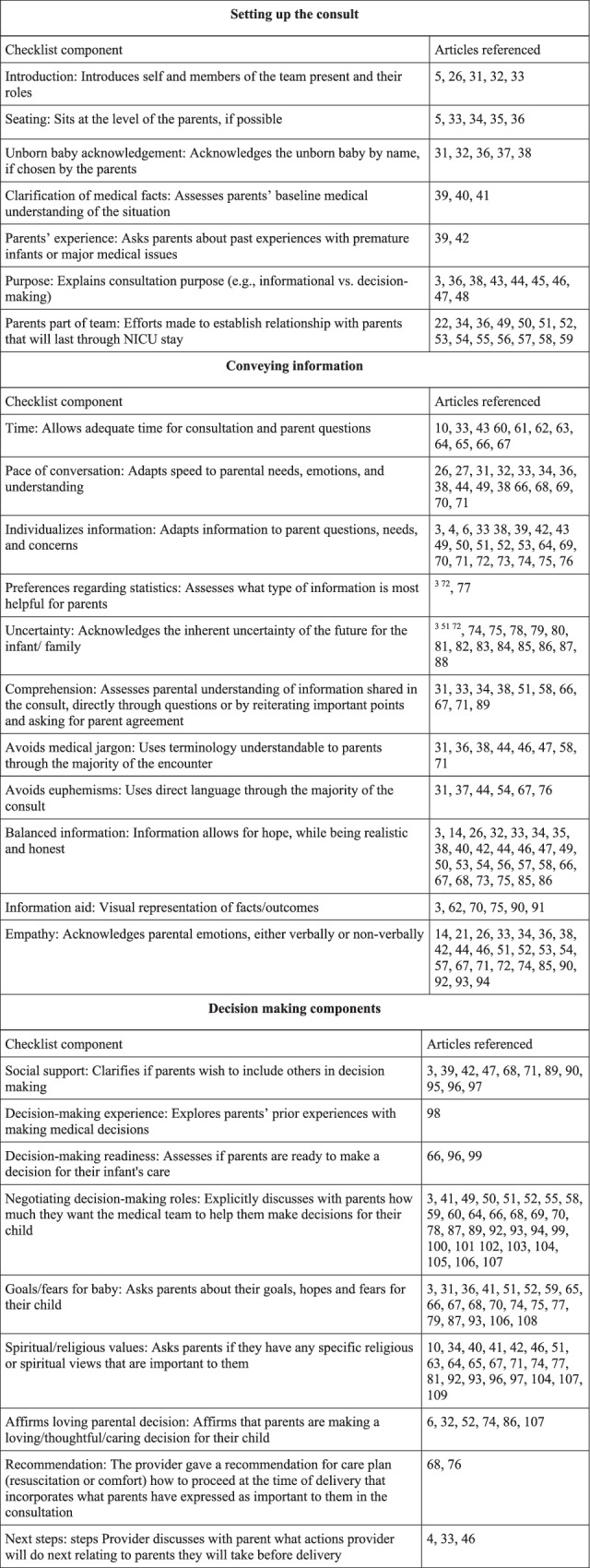
Checklist for shared decision-making. Revised checklist with articles referenced in development of each [31–109] checklist component.

References from the returned articles were cross-checked for additional relevant sources. Study team members reviewed the returned titles/abstracts for relevance and selected articles related to antenatal consultation for inclusion in the review. A deductive qualitative thematic analysis using a priori constructed categories was conducted on the remaining articles, and extracted themes were sorted into three primary categories related to SDM - setting up the consult, conveying information, and decision-making components [21]. Informed by these themes, the research team developed 34 discrete components of SDM to be included in the checklist. Component items were grouped by theme and ordered in a sequence in which they may be encountered during a consultation for ease of use. The checklist and reference articles for each item are found in Fig. 1.

### Checklist evaluation

To further refine and evaluate the reliability of the checklist, a prospective cohort of neonatologists consented to provide antenatal consultation during video-recorded simulated encounters between June 2021 and May 2022. Neonatologists at four participating centers within the United States were eligible for inclusion in the study. This study operated under the individual Institutional Review Boards and with cooperative agreements between NorthShore University HealthSystem Evanston Hospital and the other participating centers. This study was performed in accordance with the Declaration of Helsinki.

A standardized patient was conceptualized depicting a woman facing possible premature delivery at 22 0/7 weeks gestation after preterm, premature rupture of membranes. Throughout the video-recorded consultations, one of two actors portrayed the standardized patient, providing consistency in the role. Prior to the consultation, participants were given basic information about the patient and were encouraged to prepare for the session as they would in their standard practice. They were asked to assume that their center had no set guidelines regarding resuscitation of infants at 22 weeks gestational age.

### Checklist validation and statistical analysis

Checklist consistency and internal validity were assessed in an iterative process by eight independent scorers using the tool to score the video-recorded mock consultations. Scorers consisted of four attending neonatologists who participated in the initial development of the SDM checklist (experts) and four additional scorers who did not participate in checklist development (novices). Novices consisted of one attending neonatologist, two neonatology fellows, and one pediatric resident. Each scorer underwent standardized training with the study team leads. Training consisted of viewing a video-recorded consultation that was not part of the study sample and using the checklist to assess the components of that consultation as a group with direct feedback provided by one of the checklist developers. Additional examples were discussed to further clarify the intent of the items. This allowed for further clarification of the scoring process and for standardization of the interpretation of each item, following the response process of Messick’s framework [20]. Using the checklist, each scorer assessed all video-recorded mock consultations and entered scores into a REDCap electronic data capture tool hosted at the coordinating center [22, 23].

Aligning with the internal structure source of Messick’s validity framework, intraclass correlation coefficients (ICC) were calculated using a two-way random-effects model to assess inter-rater agreement for each item, subcategory, and the checklist in its entirety. A range of ICC values between 0.5 and 0.75 represented moderate reliability, which was a priori deemed to be acceptable for this relatively “moderate stakes” evaluation tool [24]. Internal consistency, or the extent of inter-relatedness of the items within the test, was calculated using Cronbach’s alpha [25]. Calculations were performed using Excel (Microsoft Inc., Redmond, WA).

Following the first round of scoring, individual checklist components with low ICC were further evaluated for clarity and reworded or removed as necessary based on study team consensus. Redundant items were eliminated or combined to further simplify the checklist. Additional training to familiarize scorers was performed. The revised checklist was then utilized to re-score the 14 original mock consultations and applied to score 8 new mock consultations.

## Results

Ultimately, 88 articles were reviewed and used to create 33 checklist items. Items were divided between three primary categories - setting up the consult, conveying information, and decision-making components. Six items were removed based on feedback from the rating team and analysis that showed poor agreement between scorers on certain items after the first round of scoring. Five items were from the “conveying information” category (addresses bias, options/choices, quality of life, parent role in the NICU, and family needs); the final item was from the “decision making” category (decision aid). The finalized checklist containing 27 items and supporting references are found in Fig. 1.

Mock consultation videos performed by 22 neonatologists were independently scored by the eight scorers, yielding 176 sets of consultation scores. Participant neonatologists represented 4 centers total. Demographic information of neonatologists providing mock consultations is reported in Table 1. Median duration of practice was 9 years (range 0–40 years). Fifty percent were male. Most (*n* = 12) performed consultations for expected extremely preterm delivery a few times per year.

**Table 1:** Characteristics of subjects performing mock consultations.

Characteristic	Category	Number of subjects
Gender	Male	11
	Female	11
Years in Practice	1–5	9
	6–10	3
	11–15	3
	16–20	0
	>20	7
Frequency Performing Extremely Preterm Consultations	1–2 times per month	1
	Once per month	2
	1–2 times every other month	1
	1–2 times every few months	1
	Once every couple of months	1
	5–8 times per year	1
	6–7 times per year	1
	5–10 times per year	1
	A few times per year	12
	A couple times per year	1

The results of mock consultation evaluation with the revised checklist are shown in Table 2. Component ICCs for the checklist were calculated. Following data analysis of the initial modified checklist, individual component item performance was assessed. The six items deemed most difficult to assess based upon lowest ICCs were removed and data analysis was repeated. Expert (ICC = 0.68) and novice (ICC = 0.52) scorers demonstrated moderate inter-rater reliability for the checklist, in keeping with the pre-defined acceptable level of reliability. Setting up components were most reliable among both expert and novice scorers, while decision making components were least reliable. Internal consistency of the checklist was high (Cronbach’s alpha = 0.93).

**Table 2:** Results from testing of modified checklist.

Modified Checklist Reliability (ICC)			
	Expert	Novice	Total
Setting Up	0.64	0.45	0.59
Conveying Info	0.58	0.34	0.48
Decision Making	0.51	0.32	0.36
SDM Process Overall	0.68	0.52	0.59
Intraclass Coefficient (ICC) Reliability			
<0.5 = Poor			
0.5–0.75 = Moderate			
0.76–0.89 = Good			
>0.9 = Excellent			

## Discussion

In this study we describe the development and testing of a checklist for assessment of the SDM process in counseling parents facing extremely preterm delivery. Using the framework described by Messick, we produced a checklist of items with a strong construct validity that demonstrated moderate reliability for the overall process of SDM in such encounters [20]. This novel checklist shows promise for helping to evaluate the shared decision-making process in future research and clinical practice.

Our initial checklist consisted of 33 items grouped into 3 primary categories - setting up the consult, conveying information, and decision-making components. Following initial testing and review with feedback from the checklist developers and scorers, five items from the “conveying information” category were removed. “Addresses bias” was determined to be difficult to assess. “Options/choices” and “parent role in the NICU” were determined to be information items, and not related to the actual practice of conveying information (included in another checklist performed concurrent to this study). “Quality of life” was felt to be included in the “uncertainty” item, as well as in the “Goals/fears for baby” and was therefore redundant. “Family needs” was similarly incorporated into the “individualizes information” item. From the “decision making” category the “decision aid” item was removed, as there was disagreement among the scorers as to what constituted a decision aid vice an information aid; the items were therefore combined. While still considered to be important and possibly included in antenatal consultations, the difficulty in assessment of these items led to their modification or exclusion from the final checklist.

Shared decision-making supports parents in making decisions with unclear consequences and when no clear answer exists, such as in the case of extreme prematurity [1]. Essential components of SDM include recognizing and acknowledging that a decision is required, knowing and understanding the best available evidence, and incorporating the patient's/family’s values and preferences into the decision [26]. It is important to consider that the parents’ expectations of the purpose of the consultation may differ from that of the counseling physician and should be assessed to ensure effective collaboration. Despite the recommendation to include SDM in antenatal counseling, no established methods have previously existed for assessing this process. Thus, this validated checklist for evaluating the discrete components of this process may serve as a valuable tool for assessing which components of SDM are most important to parents. While clinical tools exist for assessing the extent to which patients are involved in the process of decision-making, (e.g., the SDM-Q-9 and SDM-Q-Doc, and the OPTION scale) [18, 19], evaluation of these rating scales has been performed primarily in the outpatient setting and most commonly in the adult population. While they contain many similar components to our checklist, they lack some of the specificity to the antenatal consult that are uniquely important to decision making for extremely preterm infants.

This checklist may be immediately helpful in the clinical setting. Levels of instruction in communication skills are variable in neonatology training, despite its importance in caring for extremely preterm infants [11]. A validated checklist for evaluating the use of SDM when conducting antenatal consultations could improve the content of those consultations, increase the level of comfort of those providing consultations, and improve the effectiveness and efficiency of the antenatal consultation process. By providing an objective tool, learners and experienced clinicians alike can be observed conducting consultations with real or mock patients and given specific feedback related to performance.

Our study consisted of a cohort of neonatologists from four geographically distinct institutions. This geographic variation in practice sites and neonatologist characteristics may increase the generalizability of our study. Additionally, recruiting physicians with differing clinical and demographic backgrounds mirrors the circumstances often encountered in clinical practice. The use of a mock patient during simulated encounters has been shown to closely mimic real-world behavior, further adding to the strength of our findings [27–30]. The purpose of this study was not to promote a prescriptive, “how-to” plan for conducting antenatal consultations for parents facing extremely preterm delivery. Rather, the objective was to describe the creation of a checklist that may reliably assess the SDM components present in an observed consultation to aid in future research and clinical teaching efforts.

While based on literature which included studies of parental perspectives, the next steps to test the validity of this checklist involve examining if presence of behaviors in the checklist correlate to parental satisfaction. Understanding what components of antenatal consultation are important to parents facing extremely preterm birth will afford clinicians the opportunity to focus on those aspects to optimize the delivery of counseling to those families. To this end, we plan to evaluate parental attitudes toward satisfaction with SDM as it relates to extremely preterm delivery by having parents view the same video-recorded consults and provide feedback. This will allow for further validity of the tool as it will address the element of “relationship to other variables” within Messick’s framework [20]. By allowing parents to view these same mock consultations, their feedback can be compared to overall scores and individual checklist items to assess which elements of SDM are most meaningful for parents in being able to make decisions for their children. Since the goal of the antenatal consultation is to adapt to parental needs and empower them through a personalized decision-making process, ensuring that healthcare providers focus on components that are demonstrated to be more important to parents is key to effective consultations [3]. Additional studies are planned to assess parental attitudes related to satisfaction with the SDM process, preparedness to make a decision following consultation, and feelings of inclusion in the decision-making process and integration into the healthcare team (Messick’s “consequences”) [20]. Through incorporation of validated observations of counseling components using our checklist, it may be possible to better generalize what constitutes a meaningful consultation for parents facing extremely preterm birth.

## Conclusion

This novel checklist to evaluate the SDM process in antenatal counseling for parents facing extremely preterm delivery demonstrates moderate reliability among scorers of different experience levels. This may serve as a useful tool to objectively assess perinatal consultations for components that are important to parents facing extremely preterm delivery and can be used to guide training in conducting these consultations. Future investigation includes parental assessments of the same video-recorded mock consultations to provide external validation of the checklist items, and parent opinions on the quality of the consultations.

## Data Availability

All data used in this study and in the creation of this manuscript are available upon request from the corresponding author.
